# Clinical impact of first-line chemotherapy combined with immune checkpoint inhibitors for limited-stage small cell lung cancer patients: a real-world propensity score matching study

**DOI:** 10.3389/fimmu.2026.1731123

**Published:** 2026-01-21

**Authors:** Quanman Hu, Chenyi Zhou, Fei Zhao, Xiaoru Song, Yuan Ding, Xiyin Wang, Boying Wu, Huina Wang, Shuaiyin Chen, Bin Jia

**Affiliations:** 1 College of Public Health, Zhengzhou University, Zhengzhou, China; 2 First Affiliated Hospital of Zhengzhou University, Zhengzhou, China; 3 Hami Central Hospital (First Affiliated Hospital of Zhengzhou University Hami Branch), Hami, China

**Keywords:** chemotherapy, Cox proportional hazards, ICIs, LS-SCLC, prognostic, PSM

## Abstract

**Background:**

Findings from the ADRIATIC clinical trial revealed that adjuvant treatment with durvalumab following chemoradiotherapy (CRT) in limited-stage small cell lung cancer (LS-SCLC) significantly improved both overall survival (OS) and progression-free survival (PFS). However, the clinical impact remains uncertain in real-world clinical practice.

**Materials and methods:**

We gathered data of LS-SCLC patients at the First Affiliated Hospital of Zhengzhou University and conducted propensity score-matched analysis (PSM), Kaplan-Meier (K-M) method and Cox proportional hazards regression.

**Results:**

Prior to PSM, survival results demonstrated the mOS of the chemotherapy group was 20.34 months (95% confidence interval (*CI*): 18.80 - 23.57 months), whereas that of the chemotherapy + ICIs group was 26.38 months (95% *CI*: 22.97 - 38.90 months); the hazard ratio (*HR*) was 0.603 (95% *CI*: 0.413 - 0.880, *P* = 0.008, sample size: 102 vs 66). Simultaneously, the mPFS of the chemotherapy + ICIs group was also greater than that of the chemotherapy group, being 10.37 months (95% *CI*: 9.03 - 12.90 months) and 7.87 months (6.63 - 9.73 months), *HR* = 0.651 (95% *CI*: 0.457 - 0.927). After 1:1 matching for basic variables in the chemotherapy group (sample size: 66), its mOS was at 20.22 months, and mPFS was longer at 8.50 months. The multivariate analysis presented that radiotherapy, systemic immune-inflammation index (SII) > 666.29, and platelet-to-lymphocyte ratio (PLR) > 261.39 were independent prognostic factors for OS.

**Conclusion:**

These results offer reliable references for clinicians when formulating treatment strategies for LS-SCLC patients and also provide support for future clinical trials.

## 1 Introduction

Lung cancer remains the primary cause of cancer incidence and mortality worldwide, including in China. Approximately 15% of newly diagnosed lung cancer cases are classified as small cell lung cancer (SCLC) (1, 2). SCLC is a highly aggressive, poorly differentiated, high-grade neuroendocrine carcinoma. In the United States, the 2021 incidence rate was 4.7 cases per 100,000 population, with a dismal five-year overall survival (OS) rate of 12% to 30% (3). In clinical practice, SCLC is categorized into two types: extensive stage (ES)-SCLC and limited stage (LS)-SCLC (4). At diagnosis, approximately 70% of patients present with ES-SCLC, while LS-SCLC accounts for the remaining 30% (5). Unlike non-small cell lung cancer (NSCLC), therapeutic advancements for SCLC have progressed relatively slowly. Although SCLC demonstrates high initial sensitivity to chemotherapy and radiotherapy, responses are typically not durable (6). Current guidelines define curative intent as the treatment goal for LS-SCLC. The standard of care consists of concurrent thoracic radiotherapy (TRT) with 4 to 6 cycles of platinum-etoposide chemotherapy (cisplatin or carboplatin plus etoposide), followed by prophylactic cranial irradiation (PCI) for patients achieving a favorable response (7). Despite this aggressive multimodal treatment strategy, the five-year survival rate for LS-SCLC remains modest, at only 30–35% (8).

In recent years, immune checkpoint inhibitors (ICIs) have shown significant advancements in the management of ES-SCLC. Multiple clinical trials, such as IMpower133 and CASPIAN, have incontrovertibly demonstrated that the combination of chemotherapy and ICIs (atezolizumab or durvalumab) can effectively extend the survival of patients with ES-SCLC (9, 10). However, for patients with LS-SCLC, there remains insufficient clinical evidence to support the routine use of immunotherapy. Notably, several preclinical studies have indicated a potential synergistic effect between chemoradiotherapy (CRT) and ICIs (11, 12). Furthermore, findings from the ADRIATIC clinical trial revealed that adjuvant treatment with durvalumab following CRT in LS-SCLC significantly improved both OS and progression-free survival (PFS) (13). In addition, a phase 1/2 clinical trial demonstrated that the combination of concurrent CRT with pembrolizumab was well tolerated and yielded promising improvements in median OS and median PFS among patients with LS-SCLC (14). Although the above-mentioned clinical trials have established the position of ICIs in LS-SCLC, several important knowledge gaps still exist. For instance, real-world evidence in LS-SCLC chemotherapy ± ICIs controlling for radiotherapy is limited, and there is almost no ‘head-to-head’ evidence directly comparing the efficacy and safety of PD-1 inhibitors and PD-L1 inhibitors.

Immunity and inflammation assume a crucial role in the immunotherapy of SCLC. At present, several novel comprehensive immune indicators, including the lung immune prognostic index (LIPI), systemic immune-inflammation index (SII), systemic inflammation response index (SIRI), and prognostic nutrition index (PNI), have been closely linked to poor unfavorable outcomes in the immunotherapy of NSCLC and ES-SCLC (15, 16). Nevertheless, the significance of these indices in the immunotherapy of LS-SCLC remains elusive.

Since the approval of the immunotherapy for ES-SCLC in China in 2020, immunotherapy has occasionally been implemented in clinical practice for LS-SCLC patients. Consequently, we gathered data of LS-SCLC patients from 2020 to 2022 at a large-scale medical center in China. Propensity score matching (PSM) was employed to evaluate the clinical implications of the combination of chemotherapy and immunotherapy for LS-SCLC. Additionally, the treatment response and safety were assessed. A multivariate Cox regression analysis was also performed to investigate the prognostic value of these newly developed comprehensive immune and nutritional indicators in LS-SCLC patients undergoing immunotherapy.

## 2 Methods

### 2.1 Study design and patients

This retrospective study was conducted in accordance with the revised Declaration of Helsinki and received ethical clearance from the Ethics Committee of the First Affiliated Hospital of Zhengzhou University ((Ethics Approval Number: 2024-KY-0189-002, Ethics Approval Date: 2024-03-11). Patients diagnosed with SCLC who were treated at the hospital between January 2020 and April 2022 were included in the study. All cases were confirmed by cytological or histological findings and independently reviewed by the attending physician. Given the retrospective design, institutional review board approval exempted informed consent requirements, and all patient data were handled under strict confidentiality protocols.

LS-SCLC was staged per Veterans Administration Lung Study Group (VALSG) criteria (17). It should be noted that the treatment plans for all patients initially diagnosed with LS-SCLC are determined by the attending physician in accordance with the current year’s Chinese CSCO guidelines, in collaboration with a multidisciplinary team including radiation oncology and radiology.

First-line therapy consisted of etoposide-platinum chemotherapy ± ICIs (concurrent). Patients undergoing thoracic radiotherapy encompass those in the concurrent CRT or sequential CRT. The target-related regions and irradiation techniques are meticulously evaluated by professional radiation oncologists. Typically, the radiation dose is 45 Gy, administered twice daily (with an interval of at least 6 hours).Exclusion criteria included: 1–2 treatment cycles for LS-SCLC, prior malignancies, surgical intervention, and Eastern Cooperative Oncology Group performance status (ECOG PS) >1. and the follow-up period concluded until September 1, 2024.

### 2.2 Data collection and evaluation

Efficacy of first-line etoposide-platinum ± ICIs in LS-SCLC was assessed via Kaplan-Meier (K-M) analysis for PFS and OS. Tumor responses per RECIST v1.1 determined objective response rate (ORR (objective response rate) = (complete response (CR) + partial response (PR)) and disease control rate (DCR (disease control rate) = CR + PR + stable disease (SD)) from imaging assessments during treatment. As this study was a single-center retrospective design and based on real-world clinical practice, all image evaluations were “investigator-assessed” and no centralized independent image review was conducted. To ensure the consistency of the evaluations, a second investigator conducted a review.

Baseline characteristics were obtained before treatment initiation, encompassing demographic data (age, gender), smoking status, ECOG performance status, radiotherapy, TNM stage, and laboratory indicators (white blood cell count, lymphocyte count, monocyte count, neutrophil count, lactate dehydrogenase (LDH), platelets, albumin, and tumor markers). Several derived inflammatory indices were also computed, including the neutrophil-to-lymphocyte ratio (NLR), derived NLR (dNLR), lymphocyte-to-monocyte ratio (LMR), platelet-to-lymphocyte ratio (PLR), SII, SIRI, PNI, LIPI, and platelet-to-albumin ratio (PAR) (18). NLR, dNLR, LDH and tumor markers were categorized using upper limit of normal (ULN) thresholds. It is worth noting that LIPI classifies based on dNLR and LDH: good (dNLR < 3 and LDH < ULN), intermediate (dNLR ≥ 3 or LDH ≥ ULN), poor (dNLR ≥ 3 and LDH ≥ ULN). Finally, optimal cutoffs for SII, LMR, PLR, SIRI, PNI, and PAR were determined via the surv_cutpoint function, we also use the median of these indicators as cutoffs for comparison.

### 2.3 Statistical analysis

Baseline characteristics were summarized using frequencies/percentages and compared between treatment groups via χ² (categorical) or Kruskal-Wallis (continuous) tests. Survival analysis employed Kaplan-Meier curves with log-rank testing. PSM used 1:1 optimal pairing (caliper=0.05) with covariates: age, sex, smoking status, ECOG PS, primary site, radiotherapy, PCI, tumor size, and nodal metastasis. Cox regression identified OS/PFS predictors; variables with univariate *P* < 0.10 entered multivariate stepwise modeling. All analyses used R (v4.4.2) and SPSS v25.0 (*P* < 0.05 significance threshold).

## 3 Results

### 3.1 Basic characteristics

**Figure 1:**
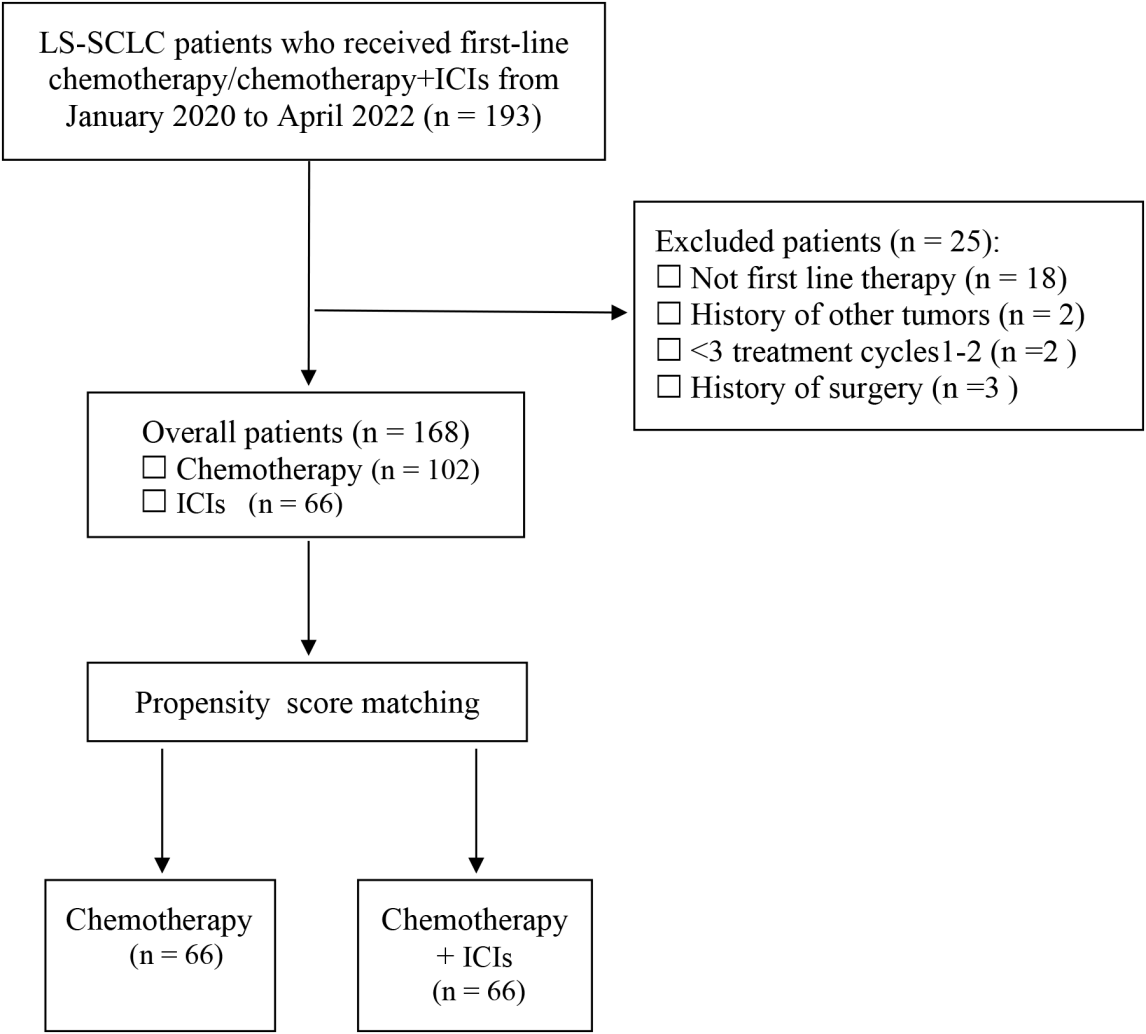
Diagram of the patient’s selection process. LS-SCLC, limited-stage small cell lung cancer; ICIs, immune checkpoint inhibitors.

Among the initial 193 patients with LS-SCLC, 168 patients were ultimately included following screening. This included 102 patients who underwent chemotherapy and 66 patients who received chemotherapy in combination with ICIs. After 1:1 PSM, a total of 66 patients were included in both the chemotherapy group and the combined chemotherapy and ICIs group (**Figure 1**). The baseline characteristics of the 168 enrolled LS-SCLC patients are summarized in **Table 1**. Specifically, 100 patients (59.52%) were under 65 years of age, 132 patients (78.57%) were male, the smoking proportion was 45.24%, and 95 patients (56.55%) had an ECOG PS of 1. The proportion of patients with a history of radiotherapy was 47.62%. Among them, 39.10% received thoracic chemoradiation, while the proportion of patients who received PCI was relatively low, at only 10.1%. The proportion of patients with lymph node metastasis was relatively high, reaching 84.52%. There was no significant difference in the proportion of the initial tumor location (left or right) and tumor size (<5 cm or ≥5 cm).

**Table 1:** Baseline characteristics before and after propensity score matching according to first-line therapy (chemotherapy/chemotherapy+ICIs).

Variables	Subcategories	n All patients (n = 168)	Before matching			After matching		
			Chemotherapy (n = 102)	Chemotherapy +ICIs (n =66)	*P*	Chemotherapy (n = 66)	Chemotherapy +ICIs (n = 66)	*P*
Age	<65	100 (59.52)	63 (61.76)	37 (56.06)	0.462	39 (59.09)	37 (56.06)	0.725
	≥65	68 (40.48)	39 (38.24)	29 (43.94)		27 (40.91)	29 (43.94)	
Sex	Male	132 (78.57)	80 (78.43)	52 (78.79)	0.956	54 (81.82)	52 (78.79)	0.662
	Female	35 (20.83)	22 (21.57)	13 (19.70)		12 (18.18)	13 (19.70)	
Smoke	NO	92 (54.76)	49 (48.04)	43 (65.15)	0.030	43 (65.15)	43 (65.15)	1.000
	YES	76 (45.24)	53 (51.96)	23 (34.85)		23 (34.85)	23 (34.85)	
ECOG PS	0	73 (43.45)	42 (41.18)	31 (46.97)	0.459	29 (43.94)	31 (46.97)	0.727
	1	95 (56.55)	60 (58.82)	35 (53.03)		37 (56.06)	35 (53.03)	
Primary site	Left	74 (44.05)	49 (48.04)	25 (37.88)	0.195	27 (40.91)	25 (37.88)	0.722
	Right	94 (55.95)	53 (51.96)	41 (62.12)		39 (59.09)	41 (62.12)	
Radiotherapy	NO	88 (52.38)	56 (54.90)	32 (48.48)	0.385	30 (45.45)	32 (48.48)	0.727
	YES	80 (47.62)	46 (45.10)	34 (51.52)		36 (54.55)	34 (51.52)	
Thoracic chemoradiation	NO	104 (61.90)	65 (63.73)	39 (59.09)	0.546	37 (56.06)	39 (59.09)	0.725
	YES	64 (39.10)	37 (36.27)	27 (40.91)		29 (43.94)	27 (40.91)	
PCI	NO	150 (89.29)	94 (92.16)	56 (84.85)	0.135	58 (87.88)	56 (84.85)	0.612
	YES	18 (10.71)	8 (7.84)	10 (15.15)		8 (12.12)	10 (15.15)	
Tumor size	<5cm	84 (50.00)	52 (50.98)	32 (48.48)	0.752	33 (50.00)	32 (48.48)	0.862
	≥5cm	84 (50.00)	50 (49.02)	34 (51.52)		33 (50.00)	34 (51.52)	
Node metastases	NO/LOCAL	26 (15.48)	17 (16.67)	9 (13.64)	0.596	9 (13.64)	9 (13.64)	1.000
	Distant	142 (84.52)	85 (83.33)	57 (86.36)		57 (86.36)	57 (86.36)	

SCLC, Small cell lung cancer; ICIs, immune checkpoint inhibitors. ECOG PS, Eastern Cooperative Oncology Group performance status. PCI, Prophylactic cranial irradiation.

Before PSM, imbalances in certain variables were observed between the chemotherapy group and the chemotherapy plus ICIs group. For example, the proportion of smokers in the chemotherapy group was 51.96%, whereas in the chemotherapy + ICIs group, it was 34.95%, with a statistically significant difference (*P* = 0.030). The proportion of patients with the primary site located on the left in the chemotherapy group was 48.04%, while in the chemotherapy + ICIs group, it was 37.88%.

Following PSM, these variables exhibited greater balance. Regarding smoking, *P* = 0.717, and for the primary site, *P* = 1.000. Additionally, **Supplementary Table S1** displays the Standardized Mean Difference (SMD) values before and after PSM. The total SMD value before PSM was 0.487, while after PSM, it decreased to 0.104. Moreover, all SMD values for the variables after PSM were below 0.1, indicating balance between the two groups. **Supplementary Figures S1**, **S2** also display the PSM results.

### 3.2 Survival analysis before and after PSM

**Figure 2:**
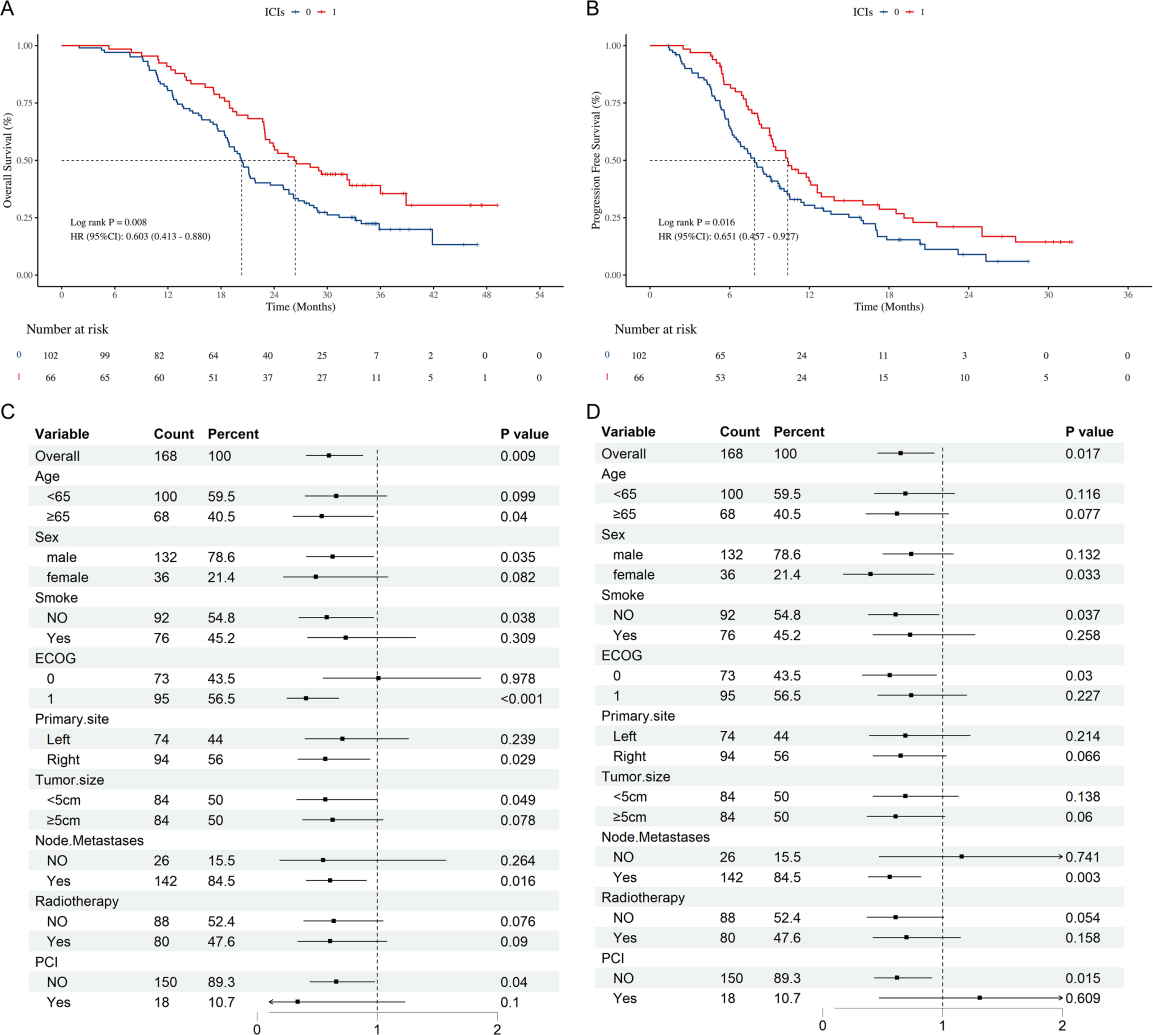
Survival outcomes of chemotherapy/chemotherapy +ICIs. **(A)** K-M curves of OS stratified by chemotherapy/chemotherapy +ICIs; **(B)** K-M curves of PFS stratified by chemotherapy/chemotherapy +ICIs; **(C)** Forest plot of subgroup analysis of OS; **(D)** Forest plot of subgroup analysis of PFS; OS, overall survival; PFS, progression free survival; ICIs, immune checkpoint inhibitors. HR, hazard ratio; CI, confidence interval; ECOG PS, Eastern Cooperative Oncology Group performance status; PCI prophylactic cranial irradiation.

Before PSM, survival analyses were performed on the chemotherapy group and the chemotherapy plus ICIs group. The results are presented in **Figure 2**. The mOS of the chemotherapy group was 20.34 months (95% confidence interval (*CI*): 18.80 - 23.57 months), whereas that of the chemotherapy + ICIs group was 26.38 months (95% CI: 22.97 - 38.90 months); the hazard ratio (*HR*) was 0.603 (95% CI: 0.413 - 0.880, *P* = 0.008, **Figure 2A**). Simultaneously, the mPFS of the chemotherapy + ICIs group was also greater than that of the chemotherapy group, being 10.37 months (95% *CI*: 9.03 - 12.90 months) and 7.87 months (6.63 - 9.73 months), respectively; *HR* = 0.651 (95% CI: 0.457 - 0.927, **Figure 2B**).

**Figure 3:**
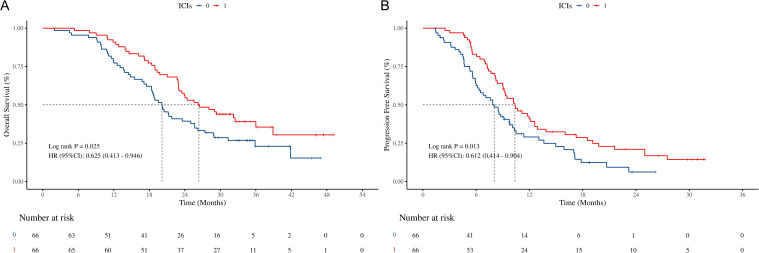
Survival outcomes of chemotherapy/chemotherapy +ICIs after PSM. **(A)** K-M curves of OS stratified by chemotherapy/chemotherapy +ICIs after PSM; **(B)** K-M curves of PFS stratified by chemotherapy/chemotherapy +ICIs after PSM; OS, overall survival; PFS, progression free survival; PSM, propensity score matching; ICIs, immune checkpoint inhibitors. HR, hazard ratio; CI, confidence interval.

Subgroup analysis results indicated that certain subgroups of variables, such as Age < 65, smoke = Yes, primary site = left, tumor size ≥ 5cm, node metastases = NO, PCI = Yes, did not derive benefit from the combination of chemotherapy and ICIs (**Figures 2C, D**). Subsequently, PSM was performed, and the survival analysis results demonstrated that after 1:1matching for some variables in the chemotherapy group, its mOS was at 20.22 months (95% CI: 18.37-26.20 months), HR = 0.625 (95% CI: 0.413 - 0.946, P = 0.025, **Figure 3A**), while its mPFS was at 8.50 months (95% CI: 7.33-10.53 months), HR = 0.612 (95% CI = 0.414 - 0.904, P = 0.013, **Figure 3B**).

### 3.3 Treatment response and related adverse events

**Figure 4:**
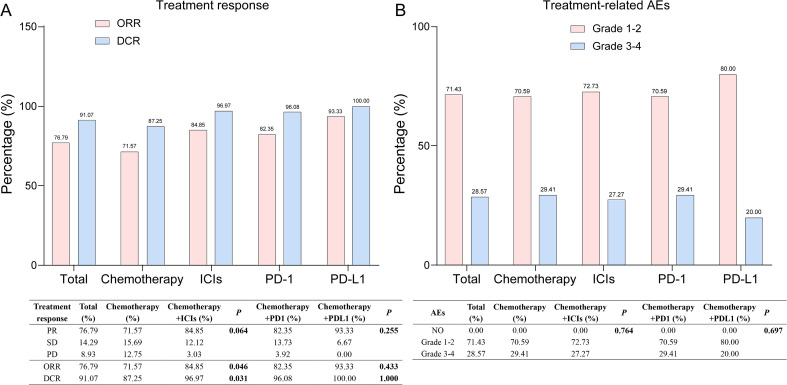
Tumor response and treatment-related AEs in all LS-SCLC patients. **(A)** Tumor response in all LS-SCLC patients. **(B)** Treatment-related AEs in all LS-SCLC patients. AEs, adverse events; LS-SCLC, limited-stage small cell lung cancer; ICIs, immune checkpoint inhibitors; PR, partial response; SD, stable disease; ORR, objective response rate; DCR, disease control rate; PD-1, programmed cell death protein 1; PD -L1, programmed cell death ligand 1.

We conducted an in-depth analysis of the treatment responses of all enrolled LS-SCLC patients. As shown in **Figure 4A**, the DCR and ORR were76.79% and 91.07%, respectively. Specifically, the DCR was 87.25% in the chemotherapy-only group and96.97% in the group receiving chemotherapy combined with ICIs, a difference that was statistically significant (*P* = 0.031). The ORR of these two groups was 71.57% and84.85%, respectively (*P* = 0.046). When compared with the chemotherapy - only group, the chemotherapy + ICIs group exhibited a higher PR rate, which was 84.85%. However, the differences in PR, SD, and PD between the two groups were not statistically significant (*P* = 0.064). Additionally, within the chemotherapy + ICIs group, there was no statistically significant difference in ORR and DCR between patients treated with programmed death - 1 (PD - 1) inhibitors and programmed death - ligand 1 (PD - L1) inhibitors (*P* > 0.05).

Regarding treatment-related adverse events (AEs), there was no statistically significant difference in the incidence of grade ≥3 AEs between the chemotherapy-only group and the chemotherapy plus ICIs group (*P* = 0.764, **Figure 4B**). Similarly, within the chemotherapy plus ICIs group, no significant difference was observed between patients treated with PD-1 and PD-L1 inhibitors (*P* = 0.697), **Figure 4B**.

### 3.4 Prognostic factors

**Table 2** comprehensively summarize the relationships among the basic characteristics, tumor markers, inflammatory indicators, and survival outcomes (OS and PFS) of patients with LS-SCLC. Regarding the univariate analysis with PFS as the clinical endpoint, the data presented in **Table 2** reveal that in LS-SCLC patients, SII > 535.81 (*HR* = 0.49, 95% *CI*: 0.28 - 0.88, *P* = 0.016), PLR > 87.12 (*HR* = 0.29, 95% *CI*: 0.12 - 0.73, *P* = 0.008), SIRI > 0.98 (*HR* = 0.46, 95% *CI*: 0.26 - 0.83, *P* = 0.010), and PAR > 2.80 (*HR* = 0.37, 95% *CI*: 0.14 - 0.95, *P* = 0.038) were associated with an improved PFS. Nevertheless, in the multivariate analysis, none of these factors emerged as independent prognostic factors for PFS.

**Table 2:** Univariate and multivariate analyses of overall survival in LS-SCLC patients who received first-line chemotherapy +ICIs.

Variables	Subcategories	OS-univariate		OS-multivariate		PFS-univariate		PFS-multivariate	
		*P*	HR (95%CI)	*P*	HR (95%CI)	*P*	HR (95%CI)	*P*	HR (95%CI)
Age	<65								
	≥65	0.930	0.93 (0.50 ~ 1.73)			0.6255	1.15 (0.66 ~ 2.00)		
Sex	Male								
	Female	0.615	1.21 (0.58 ~ 2.55)			0.267	0.68 (0.34 ~ 1.35)		
Smoke	NO								
	YES	0.222	1.48 (0.79 ~ 2.79)			0.738	1.10 (0.62 ~ 1.96)		
ECOG PS	0								
	1	0.509	1.23 (0.66 ~ 2.28)			0.541	1.19 (0.68 ~ 2.06)		
Primary site	Left								
	Right	0.386	0.76 (0.41 ~ 1.42)			0.473	0.81 (0.45 ~ 1.44)		
Radiotherapy	NO								
	YES	0.044	0.38 (0.15 ~ 0.98)	0.013	0.30 (0.11 ~ 0.78)	0.974	1.01 (0.58 ~ 1.75)		
Tumor size	< 5cm								
	≥5cm	0.336	1.35 (0.73 ~ 2.51)			0.618	0.87 (0.50 ~ 1.51)		
Node metastases	NO/LOCAL								
	Distant	0.487	1.40 (0.55 ~ 3.57)			0.510	0.78 (0.36 ~ 1.65)		
ICIs	PD-1								
	PD-L1	0.931	1.03 (0.49 ~ 2.17)			0.792	1.09 (0.57 ~ 2.09)		
LDH	≤245								
	>245	0.104	0.52 (0.23 ~ 1.15)			0.343	0.71 (0.35 ~ 1.44)		
CEA	≤5								
	>5	0.789	1.11 (0.51 ~ 2.45)			0.108	0.51 (0.23 ~ 1.16)	0.113	0.51 (0.23 ~ 1.17)
Cyfran21-1	≤3.3								
	>3.3	0.872	1.07 (0.46 ~ 2.48)			0.496	1.28 (0.63 ~ 2.57)		
CA125	≤35								
	>35	0.402	0.74 (0.36 ~ 1.51)			0.826	1.07 (0.57 ~ 2.03)		
CA19-9	≤37								
	>37	0.269	1.73 (0.66 ~ 4.55)			0.416	1.48 (0.58 ~ 3.81)		
CA72-4	≤7								
	>7	0.693	0.75 (0.18 ~ 3.15)			0.225	0.41 (0.10 ~ 1.72)		
NSE	≤16.3								
	>16.3	0.020	0.48 (0.26 ~ 0.89)	0.074	0.55 (0.29 ~ 1.06)	0.267	0.72 (0.41 ~ 1.28)		
SII	≤666.29								
	>666.29	0.078	0.56 (0.30 ~ 1.07)	0.038	0.43 (0.20 ~ 0.95)	0.060	0.58 (0.33 ~ 1.02)		
NLR	≤4								
	>4	0.199	0.63 (0.31 ~ 1.28)			0.935	0.98 (0.53 ~ 1.78)		
dNLR	≤3								
	>3	0.339	0.66 (0.28 ~ 1.56)			0.171	0.59 (0.27 ~ 1.26)		
LIPI	Good								
	Intermediate	0.041	0.34 (0.12 ~ 0.95)			0.315	0.69 (0.34 ~ 1.41)		
	Poor	0.818	0.89 (0.35 ~ 2.30)			0.439	0.69 (0.27 ~ 1.77)		
LMR	≤5.26								
	>5.26	0.267	0.51 (0.16 ~ 1.67)			0.401	0.67 (0.27 ~ 1.70)		
PLR	≤261.39								
	>261.39	0.093	1.86 (0.90 ~ 3.85)	0.024	2.75 (1.14 ~ 6.61)	0.779	0.90 (0.44 ~ 1.86)		
SIRI	≤2.95								
	>2.95	0.148	0.50 (0.19 ~ 1.28)			0.233	1.55 (0.75 ~ 3.21)		
PAR	≤4.65								
	>4.65	0.101	0.59 (0.31 ~ 1.11)			0.044	0.54 (0.29 ~ 0.98)	0.069	0.55 (0.29 ~ 1.05)
PNI	≤179								
	>179	0.094	0.56 (0.28 ~ 1.10)			0.204	0.66 (0.35 ~ 1.25)		

OS, overall survival; PFS, progression free survival; ICIs, immune checkpoint inhibitors. HR, hazard ratio; CI, confidence interval; ES, extensive-stage; PD-1, programmed cell death protein 1; PD-L1, programmed cell death ligand 1; ECOG PS, Eastern Cooperative Oncology Group performance status; LDH, lactate dehydrogenase; NLR, neutrophil-to-lymphocyte ratio; dNLR, derived Neutrophil to Lymphocyte Ratio; LIPI, lung immune prognostic index; LMR, lymphocyte to monocyte ratio; PLR, platelet to lymphocyte ratio; PAR, platelet to albumin ratio; PNI, prognostic nutrition index; SII, systemic immune-inflammation index; SIRI, systemic inflammation response index; CEA, carcinoembryonic antigen; NSE, Neuron-specific enolase; CA125, Cancer antigen 125; CA153, Carbohydrate antigen 15-3; Cyfran 21-1, cytokeratin 19 fragments.

**Figure 5:**
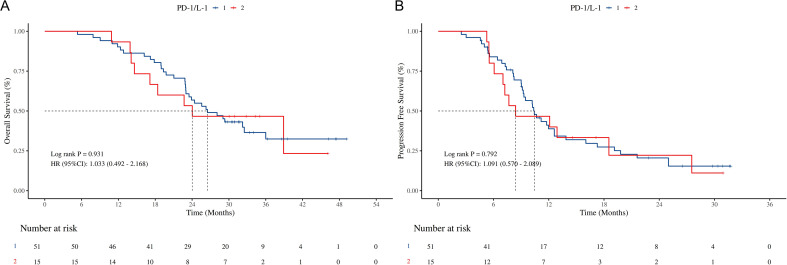
Survival outcomes of different ICIs (PD-1/PD-L1). **(A)** K-M curves of OS stratified by different ICIs (PD-1/PD-L1); **(B)** K-M curves of PFS stratified by different ICIs (PD-1/PD-L1); OS, overall survival; PFS, progression free survival; ICIs, immune checkpoint inhibitors. HR, hazard ratio; CI, confidence interval; PD-1, programmed cell death protein 1; PD-L1, programmed cell death ligand 1.

For the univariate analysis of clinical outcome as OS, the results in **Table 2** indicate that Radiotherapy (*HR* = 0.38, 95% *CI*: 0.15 - 0.98, *P* = 0.044), NSE > 16.3 (*HR* = 0.48, 95% *CI*: 0.26 - 0.89, *P* = 0.020), and LIPI = intermediate (*HR* = 0.34, 95% *CI*: 0.12 - 0.95, *P* = 0.041) were associated with improved OS in LS-SCLC patients. However, in the multivariate analysis, Radiotherapy (*HR* = 0.30, 95% *CI*: 0.11 - 0.78, *P* = 0.013), SII > 666.29 (*HR* = 0.43, 95% *CI*: 0.20 - 0.95, *P* = 0.038), and PLR > 261.39 (*HR* = 2.75, 95% *CI*: 1.14 - 6.61, *P* = 0.024) were independent prognostic factors for OS. In addition, multivariate analysis of several derived inflammatory indices with the median as the cutoff point showed that radiotherapy, SII > 663.69 and NSE > 16.3 were independent prognostic factors for OS (**Supplementary Table S2**).Furthermore, a survival analysis was performed on LS-SCLC patients in the chemotherapy plus ICIs group who underwent different types of ICIs treatment (PD-1/PD-L1). The findings are presented in **Figure 5**. No statistically significant differences were observed in OS and PFS between the PD-1 group and the PD-L1 group (OS: *HR*: 1.033 95% *CI*: 0.492 - 2.168, *P* = 0.931; PFS: *HR*: 1.091, 95% *CI*: 0.579 - 2.089, *P* = 0.792).

## 4 Discussion

Drawing upon high-quality evidence, encompassing systematic reviews, guidelines, and meta-analyses, the current standard treatment for LS-SCLC involves CCRT with platinum and etoposide (19–21). Patients who exhibit an intermittent disease response to this treatment are considered suitable candidates for PCI, as it can significantly enhance the three-year survival rate (22). Nevertheless, the effectiveness of these standard treatment modalities remains constrained. At present, chemotherapy combined with ICIs is recommended as the standard first-line treatment for ES-SCLC in the United States, Europe, China, and other countries (23–25). Prior to the ADRIATIC trial, there was a lack of evidence to support the use of immunotherapy in LS-SCLC (13). Demonstrating that immunotherapy can notably improve OS and PFS, it holds the potential to reshape the treatment paradigm of LS-SCLC. The approval of durvalumab for LS-SCLC by both the US Food and Drug Administration (FDA) and the China National Medical Products Administration (NMPA) represents a significant milestone (26, 27). Consequently, prior to the widespread clinical implementation of ICIs in combination with CCRT or chemotherapy alone, it is of great significance to explore their efficacy, safety, and the prognostic biomarkers associated with the combined treatment approach.

In this retrospective study, data were collected from patients diagnosed with LS-SCLC who received treatment at the First Affiliated Hospital of Zhengzhou University from 2020 to 2022, followed by follow-up evaluations. K-M curve analyses, conducted both before and after PSM, indicated that patients with LS-SCLC who were treated with a combination of chemotherapy and ICIs experienced improved mOS and mPFS compared to those treated with chemotherapy alone. It is worth noting that, unlike the ADRITIC trial, this study did not make CRT a mandatory requirement. Moreover, In addition, both therapies maintained acceptable safety profiles, and there was no statistically significant difference in the rates of grade ≥3 adverse events between the two groups. PSM effectively minimized the disparities in baseline variables between the two groups. Consequently, following PSM, alterations were observed in the mOS and mPFS of the chemotherapy group. This could potentially be attributed to the reduction of potential confounding factors achieved by PSM (28).

Prior to this, several clinical trials have also underscored the importance of integrating CRT with ICIs A single-center, open-label phase 1/2 clinical trial reported that the concurrent administration of CRT and pembrolizumab in patients with LS-SCLC was well tolerated. Among the 40 enrolled patients, there were only three grade 4 events, and the incidence of pneumonia was 15%. At a median follow-up of 23.1 months, the mPFS was 19.7 months (14). Another phase 3 clinical trial demonstrated that consolidation treatment with durvalumab administered every 4 weeks during CRT significantly extended the mPFS and mOS without elevating the incidence of grade 3/4 AEs such as pneumonia (3.1% vs 2.6%) (13). Conversely, the STIMULI trial showed that consolidation immunotherapy with ipilimumab plus nivolumab after CRT in LS-SCLC patients did not enhance PFS compared to the control group and failed to reach the primary endpoint. This might be attributed to the high dropout rate (35%) induced by severe AEs during the trial (29). In conclusion, these studies indicate that the combination of CRT and ICIs in the treatment of LS-SCLC patients can significantly improve OS and PFS compared to CRT alone, with favorable tolerability.

It is noteworthy that during the collection of information on LS-SCLC cases, some patients presented with complex conditions and did not adhere strictly to the drug recommendations outlined in the Chinese CSCO guidelines. Clinicians frequently tailored medical strategies according to the actual circumstances. Consequently, a significant number of LS-SCLC patients did not undergo strict concurrent or sequential CRT, but rather received chemotherapy alone. The reasons for declining chest radiotherapy can be multifaceted, encompassing concerns regarding severe side effects associated with combined treatments, pulmonary fibrosis, and economic constraints. Moreover, an even smaller proportion of LS-SCLC patients received sequential CRT treatment. The most plausible explanation is the complexity of the treatment process, wherein patients often need to be transferred to the radiology department for treatment, and the treatment cycle is relatively protracted. In light of these considerations, we adjusted the inclusion criteria for SCLC patients, including only LS-SCLC patients who received chemotherapy and/or immunotherapy. CRT treatment was not incorporated as an inclusion criterion; instead, it was regarded as one of the covariates to be balanced in PSM. Such an analysis can offer more practical recommendations for clinicians in the real world when formulating treatment strategies for LS-SCLC patients. Nevertheless, in the treatment of stage III NSCLC, consolidation treatment with durvalumab following CRT could significantly enhance PFS (30). Subsequently, expert consensus also advocated the combination of durvalumab and CRT in NSCLC (31, 32). Likewise, the real-world study (SOLUTION) reported concordant findings (33), presumably due to the fact that the combination of radiotherapy and ICIs could induce alterations in the tumor immune microenvironment within the body (34). In the treatment of SCLC, it is essential to compare the clinical impact of CRT combined with ICIs against that of CRT alone in the future.

Regarding the combination of chemotherapy with various types of ICIs, our findings demonstrate that there are no statistically significant disparities in OS, PFS, and AEs between PD-1 inhibitors and PD-L1 inhibitors. In the treatment of ES-SCLC with chemotherapy in combination with ICIs, a network meta-analysis based on randomized controlled trials reveals that the efficacy and safety of PD-L1 inhibitors in combination with chemotherapy are comparable to those of PD-1 inhibitors combined with chemotherapy (35). Nevertheless, in NSCLC, treatment regimens incorporating anti-PD-1 agents confer better OS benefits compared to anti-PD-L1 therapy (36). Considering these inconsistent results, for LS-SCLC, with the clinical approval of durvalumab, further research is warranted in the future to elucidate the differences between PD-1 and PD-L1 when combined with chemotherapy.

Conventional factors, including smoking habits, gender, age, ECOG and TNM staging, along with other clinical indicators, are associated with the prognosis of SCLC (37). Systemic inflammation also exerts a crucial influence on tumor promotion and progression. Thus, it is plausible that markers of systemic inflammation are linked to the prognosis of SCLC. At present, certain novel systemic immune indicators, such as LIPI, can serve as prognostic factors for ES-SCLC in first-line chemotherapy and immunotherapy (38). Similarly, in studies of LS-SCLC without differentiating treatment modalities, the LIPI stratification results are key determinants affecting the prognosis of LS-SCLC patients (39). Our findings indicate that in LS-SCLC patients who received only chemotherapy plus ICIs, LIPI cannot be utilized as a prognostic factor, whereas the SII and PLR can be employed as independent predictors of OS. SII is a comprehensive immune parameter that incorporates neutrophils, lymphocytes, and platelets. Typically, an elevation in neutrophil levels, an increase in platelet count, and a decline in lymphocytes contribute to an increase in SII. A meta-analysis exploring the prognostic implications of SII in SCLC revealed a significant correlation between high SII and poor OS. Nevertheless, subgroup analysis indicated that different cut-off values of SII and the adjustment of confounding factors could influence the outcomes. In studies where the SII cut-off was set at < 700, high SII was not significantly associated with poor OS, with an odds ratio (OR) of 1.54 (95% CI: 0.83 - 2.85). Hence, multiple factors may account for the discrepancies between the findings of this study and those of the meta-analysis, including variations in cut-off values, adjustment for confounding variables, and differences in treatment modalities (40). PLR represents a composite outcome of platelets and lymphocytes, concurrently reflecting the equilibrium between the inflammatory response and the adaptive immune response. Our research findings demonstrate that a high PLR is correlated with poor OS. This is in accordance with the results of a meta-analysis, which reveals a notable decrease in OS among patients with LS-SCLC in the high PLR group (41). Our research is distinguished by its concentrated examination of LS-SCLC patients who received concurrent treatment with chemotherapy and immunotherapy.

The tumor microenvironment, consisting of the vascular system, extracellular matrix, and inflammatory cells, assumes a pivotal role in cancer progression. An increased PLR implies a surge in the inflammatory response, and it is accompanied by the regulation of immune cells and vascular signaling within the tumor microenvironment. Consequently, calculating the PLR prior to initiating chemotherapy combined with immunotherapy in LS-SCLC patients can aid in identifying those who are likely to have a poor prognosis.

Our research has certain limitations that warrant attention. One key limitation is the currently limited number of patients with LS-SCLC who underwent combined chemotherapy and immunotherapy (n = 66). This limited sample size may undermine both the statistical power and the generalizability of the findings. Additionally, the relatively wide 95% CI may give rise to concerns regarding the precision of the estimates. Secondly, the relatively short follow-up duration could have an impact on the outcomes. By the conclusion of the follow-up period, some patients had not yet reached the primary clinical endpoints, namely OS and PFS. Furthermore, given that durvalumab has been approved for the treatment of LS-SCLC for a relatively brief period, future research will necessitate a larger number of cases and more extended follow-up intervals to obtain more comprehensive and reliable data. Thirdly, owing to the retrospective nature of this study, despite the use of PSM to balance the baseline characteristics between the two treatment groups, there remains a possibility that some potential confounding factors may still be present.

## Conclusion

In summary, our results indicate that the combination of first-line chemotherapy and ICIs can significantly improve OS and PFS in patients with LS-SCLC, while maintaining an acceptable safety profile. Moreover, radiotherapy, PLR > 261.39, and SII > 666.29, emerge as independent predictors of prognosis in LS-SCLC patients receiving combined treatment modalities. These results offer reliable references for clinicians when formulating treatment strategies for LS-SCLC patients and also provide support for future clinical trials. It should be noted that, owing to the retrospective study design, limited follow - up, and other factors, these findings should be referred to with caution.

## Data Availability

The original contributions presented in the study are included in the article/**Supplementary Material**. Further inquiries can be directed to the corresponding authors.
