# Supplementary material for: Clinical impact of first-line chemotherapy combined with immune checkpoint inhibitors for limited-stage small cell lung cancer patients: a real-world propensity score matching study
Source: Front Immunol. 2026 Jan 21;17:1731123. doi: 10.3389/fimmu.2026.1731123 (PMC12867874; doi:10.3389/fimmu.2026.1731123)
Supplement: Supplementary file 1 [file DataSheet1.docx]

Supplementary material


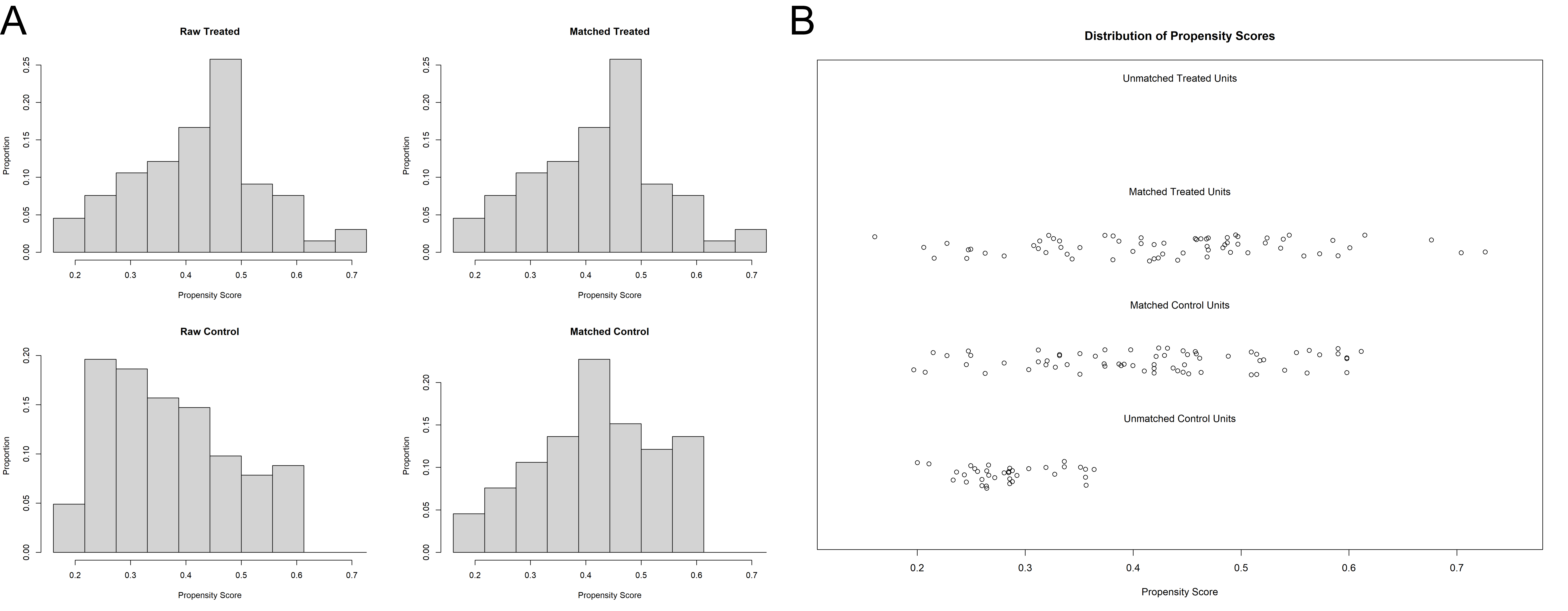


Figure S1 Comparison of data before and after propensity score match (PSM)


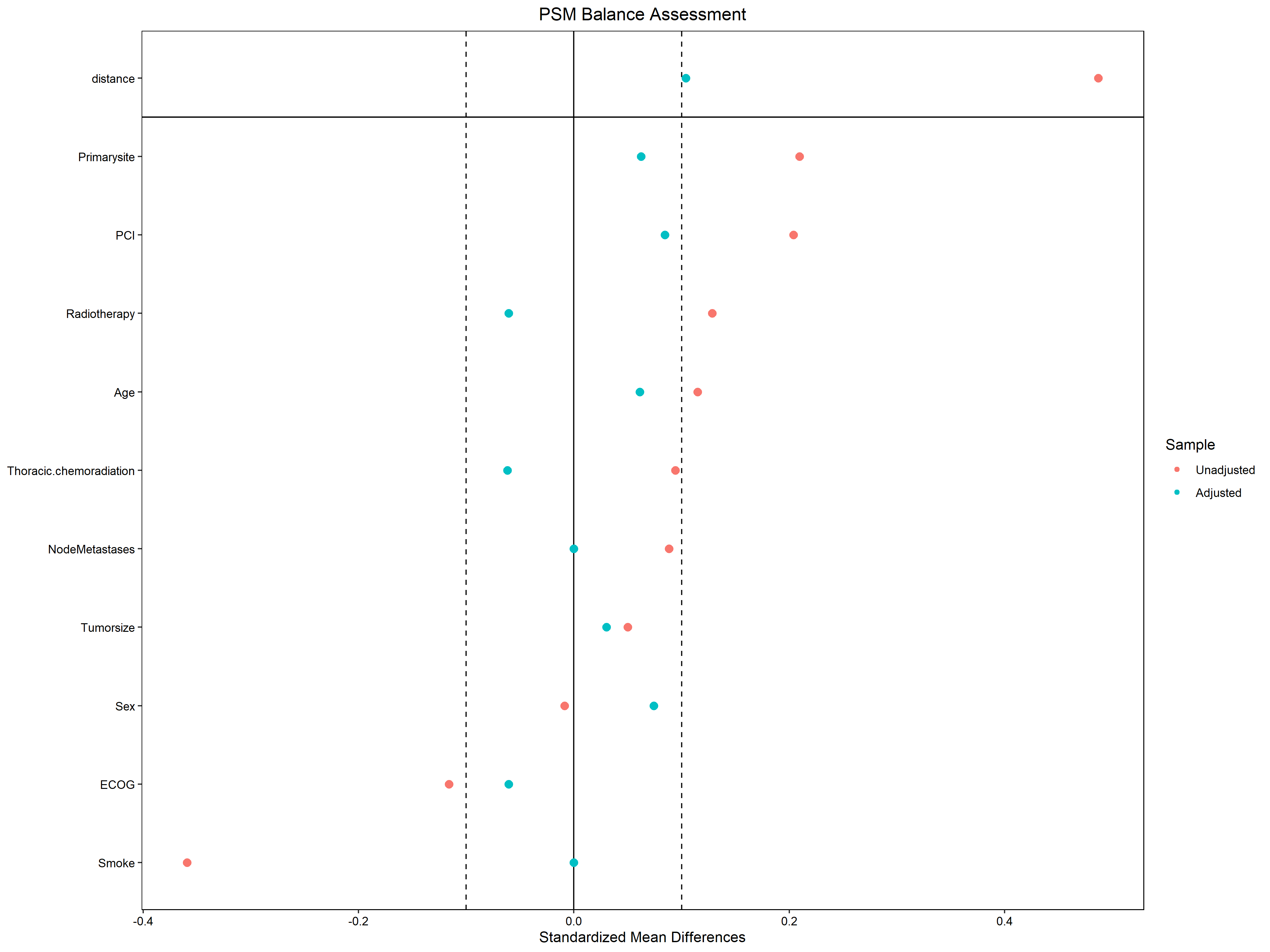


Figure S2 Love plot of before and after propensity score match (PSM)

Table S1 the SMD of different variables before and after PSM

| Variable | SMD -before PSM | SMD -after PSM |
| --- | --- | --- |
| Overall | 0.487 | 0.104 |
| Age | 0.057 | 0.030 |
| Sex | -0.004 | 0.030 |
| Smoke | -0.171 | 0.000 |
| ECOG PS | -0.058 | -0.030 |
| Primary site | 0.102 | 0.030 |
| Tumor size | 0.025 | 0.015 |
| Node metastases | 0.030 | 0.000 |
| Radiotherapy | 0.064 | -0.030 |
| Thoracic.chemoradiation | 0.046 | -0.030 |
| PCI | 0.073 | 0.030 |

Note: SMD, standardized differences; ECOG PS, Eastern Cooperative Oncology Group performance status.

**Table S2 Univariate and multivariate analyses of overall survival and progression free survival in LS-SCLC patients who received first-line chemotherapy +ICIs (cut-off value using median of SII, LMR, PLR, SIRI, PAR and PNI)**

| Variables |  |  | OS-Univariate |  | OS-Multivariate |  | PFS-Univariate |  | PFS-Multivariate |
| --- | --- | --- | --- | --- | --- | --- | --- | --- | --- |
|  |  | *P* | HR (95%CI) | *P* | HR (95%CI) | *P* | HR (95%CI) | *P* | HR (95%CI) |
| Age | <65 |  |  |  |  |  |  |  |  |
|  | ≥65 | 0.819 | 0.93 (0.50 ~ 1.73) |  |  | 0.6255 | 1.15 (0.66 ~ 2.00) |  |  |
| Sex | Male |  |  |  |  |  |  |  |  |
|  | Female | 0.615 | 1.21 (0.58 ~ 2.55) |  |  | 0.267 | 0.68 (0.34 ~ 1.35) |  |  |
| Smoke | NO |  |  |  |  |  |  |  |  |
|  | YES | 0.222 | 1.48 (0.79 ~ 2.79) |  |  | 0.738 | 1.10 (0.62 ~ 1.96) |  |  |
| ECOG  PS | 0 |  |  |  |  |  |  |  |  |
|  | 1 | 0.509 | 1.23 (0.66 ~ 2.28) |  |  | 0.541 | 1.19 (0.68 ~ 2.06) |  |  |
| Primary  site | Left |  |  |  |  |  |  |  |  |
|  | Right | 0.386 | 0.76 (0.41 ~ 1.42) |  |  | 0.473 | 0.81 (0.45 ~ 1.44) |  |  |
| Radiotherapy | NO |  |  |  |  |  |  |  |  |
|  | YES | 0.061 | 0.55 (0.29 ~ 1.03) | 0.015 | 0.32 (0.13 ~ 0.81) | 0.974 | 1.01 (0.58 ~ 1.75) |  |  |
| Tumor size | < 5cm |  |  |  |  |  |  |  |  |
|  | ≥5cm | 0.336 | 1.35 (0.73 ~ 2.51) |  |  | 0.618 | 0.87 (0.50 ~ 1.51) |  |  |
| Node  metastases | NO/LOCAL |  |  |  |  |  |  |  |  |
|  | Distant | 0.487 | 1.40 (0.55 ~ 3.57) |  |  | 0.510 | 0.78 (0.36 ~ 1.65) |  |  |
| ICIs | PD-1 |  |  |  |  |  |  |  |  |
|  | PD-L1 | 0.931 | 1.03 (0.49 ~ 2.17) |  |  | 0.792 | 1.09 (0.57 ~ 2.09) |  |  |
| LDH | ≤245 |  |  |  |  |  |  |  |  |
|  | >245 | 0.104 | 0.52 (0.23 ~ 1.15) |  |  | 0.343 | 0.71 (0.35 ~ 1.44) |  |  |
| CEA | ≤5 |  |  |  |  |  |  |  |  |
|  | >5 | 0.789 | 1.11 (0.51 ~ 2.45) |  |  | 0.108 | 0.51 (0.23 ~ 1.16) | 0.113 | 0.51 (0.23 ~ 1.17) |
| Cyfran21 - 1 | ≤3.3 |  |  |  |  |  |  |  |  |
|  | >3.3 | 0.872 | 1.07 (0.46 ~ 2.48) |  |  | 0.496 | 1.28 (0.63 ~ 2.57) |  |  |
| CA125 | ≤35 |  |  |  |  |  |  |  |  |
|  | >35 | 0.402 | 0.74 (0.36 ~ 1.51) |  |  | 0.826 | 1.07 (0.57 ~ 2.03) |  |  |
| CA19-9 | ≤37 |  |  |  |  |  |  |  |  |
|  | >37 | 0.269 | 1.73 (0.66 ~ 4.55) |  |  | 0.416 | 1.48 (0.58 ~ 3.81) |  |  |
| CA72-4 | ≤7 |  |  |  |  |  |  |  |  |
|  | >7 | 0.693 | 0.75 (0.18 ~ 3.15) |  |  | 0.225 | 0.41 (0.10 ~ 1.72) |  |  |
| NSE | ≤16.3 |  |  |  |  |  |  |  |  |
|  | >16.3 | **0.020** | 0.48 (0.26 ~ 0.89) | 0.003 | 0.25 (0.10 ~ 0.62) | 0.267 | 0.72 (0.41 ~ 1.28) |  |  |
| SII | ≤663.69 |  |  |  |  |  |  |  |  |
|  | >663.69 | 0.058 | 0.54 (0.28 ~ 1.02) | 0.030 | 0.39 (0.16 ~ 0.91) | 0.225 | 0.70 (0.40 ~ 1.24) |  |  |
| NLR | ≤4 |  |  |  |  |  |  |  |  |
|  | >4 | 0.199 | 0.63 (0.31 ~ 1.28) |  |  | 0.935 | 0.98 (0.53 ~ 1.78) |  |  |
| dNLR | ≤3 |  |  |  |  |  |  |  |  |
|  | >3 | 0.339 | 0.66 (0.28 ~ 1.56) |  |  | 0.171 | 0.59 (0.27 ~ 1.26) |  |  |
| LIPI | Good |  |  |  |  |  |  |  |  |
|  | Intermediate | 0.041 | 0.34 (0.12 ~ 0.95) |  |  | 0.315 | 0.69 (0.34 ~ 1.41) |  |  |
|  | Poor | 0.818 | 0.89 (0.35 ~ 2.30) |  |  | 0.439 | 0.69 (0.27 ~ 1.77) |  |  |
| LMR | ≤3.02 |  |  |  |  |  |  |  |  |
|  | >3.02 | 0.515 | 1.23 (0.66 ~ 2.27) |  |  | 0.358 | 1.30 (0.75 ~ 2.25) |  |  |
| PLR | ≤159.65 |  |  |  |  |  |  |  |  |
|  | >159.65 | 0.857 | 1.06 (0.57 ~ 1.96) |  |  | 0.565 | 1.18 (0.68 ~ 2.04) |  |  |
| SIRI | ≤1.34 |  |  |  |  |  |  |  |  |
|  | >1.34 | 0.981 | 0.99 (0.54 ~ 1.83) |  |  | 0.143 | 0.66 (0.38 ~ 1.15) | 0.076 | 0.58 (0.32 ~ 1.06) |
| PAR | ≤5.72 |  |  |  |  |  |  |  |  |
|  | >5.72 | 0.840 | 0.94 (0.51 ~ 1.74) |  |  | 0.272 | 0.73 (0.42 ~ 1.28) |  |  |
| PNI | ≤230 |  |  |  |  |  |  |  |  |
|  | >230 | 0.716 | 1.12 (0.61 ~ 2.07) |  |  | 0.842 | 1.06 (0.61 ~ 1.84) |  |  |

Note: OS, overall survival; PFS, progression free survival; ICIs, immune checkpoint inhibitors. HR, hazard ratio; CI, confidence interval; ES, extensive-stage; PD-1, programmed cell death protein 1; PD-L1, programmed cell death ligand 1; ECOG PS, Eastern Cooperative Oncology Group performance status;

LDH, lactate dehydrogenase; NLR, neutrophil-to-lymphocyte ratio; dNLR, derived Neutrophil to Lymphocyte Ratio; LIPI, lung immune prognostic index; LMR, lymphocyte to monocyte ratio; PLR, platelet to lymphocyte ratio; PAR, platelet to albumin ratio; PNI, prognostic nutrition index; SII, systemic immune-inflammation index; SIRI, systemic inflammation response index; CEA, carcinoembryonic antigen; NSE, Neuron-specific enolase; CA125, Cancer antigen 125; CA153, Carbohydrate antinegen 15-3; Cyfran21 - 1, cytokeratin 19 fragments
